# Measuring health-related quality of life in chronic obstructive pulmonary disease: properties of the EQ-5D-5L and PROMIS-43 short form

**DOI:** 10.1186/1471-2288-14-78

**Published:** 2014-06-16

**Authors:** Fang-Ju Lin, A Simon Pickard, Jerry A Krishnan, Min J Joo, David H Au, Shannon S Carson, Suzanne Gillespie, Ashley G Henderson, Peter K Lindenauer, Mary Ann McBurnie, Richard A Mularski, Edward T Naureckas, William M Vollmer, Todd A Lee

**Affiliations:** 1 University of Illinois at Chicago, Chicago, IL, USA; 2 University of Illinois Hospital & Health Sciences System, Chicago, IL, USA; 3 VA Puget Sound Health Care System and University of Washington, Seattle, WA, USA; 4 University of North Carolina, Chapel Hill, NC, USA; 5 Kaiser Permanente Northwest Center for Health Research, Portland, OR, USA; 6 Baystate Medical Center, Springfield, MA, USA; 7 Tufts University School of Medicine, Boston, MA, USA; 8 University of Chicago, Chicago, IL, USA; 9 Department of Pharmacy Systems, Outcomes and Policy, College of Pharmacy, University of Illinois at Chicago, 833 South Wood St., M/C 886, Chicago, IL 60612, USA

**Keywords:** COPD, EQ-5D-5L, PROMIS, Psychometric properties

## Abstract

**Background:**

The Patient Reported Outcomes Measurement Information System 43-item short form (PROMIS-43) and the five-level EQ-5D (EQ-5D-5L) are recently developed measures of health-related quality of life (HRQL) that have potentially broad application in evaluating treatments and capturing burden of respiratory-related diseases. The aims of this study were: (1) to examine their psychometric properties in patients with chronic obstructive pulmonary disease (COPD), and (2) to identify dimensions of HRQL that differ and do not differ by lung function.

**Methods:**

We conducted a multi-center, cross-sectional study (“COPD Outcomes-based Network for Clinical Effectiveness & Research Translation” [CONCERT]). We analyzed patients who met spirometric criteria for COPD, and completed EQ-5D-5L and PROMIS questionnaires. Disease severity was graded based on the Global Initiative for Chronic Obstructive Lung Disease (GOLD) classification. Pulmonary function test, PROMIS-43, EQ-5D (index score and EQ-Visual Analog Scale [EQ-VAS]), six minute walk test (6MWT), and three dyspnea scales (mMRC, Borg, FACIT-Dyspnea) were administered. Validity and reliability of EQ-5D-5L and PROMIS-43 were examined, and differences in HRQL by GOLD grade were assessed.

**Results:**

Data from 670 patients with COPD were analyzed (mean age 68.5 years; 58% male). More severe COPD was associated with more problems with mobility, self-care and usual activities (all p-values <0.01) according to EQ-5D-5L. Related domains on EQ-5D-5L, PROMIS and clinical measures were moderately (r = 0.30-0.49) to strongly (r ≥ 0.50) correlated. A statistically significant trend of decreasing HRQL with more severe lung functions was observed for EQ-5D-5L index scores, EQ-VAS scores, and PROMIS physical function and social roles.

**Conclusions:**

Results supported the validity of EQ-5D-5L and PROMIS-43 in COPD patients, and indicate that physical function and social activities decrease with level of lung function by GOLD grade, but not pain, mental health, sleep or fatigue as reported by patients.

## Background

Chronic obstructive pulmonary disease (COPD) is one of the leading causes of death worldwide, and affects 9-10% of adults aged 40 years or older [1,2]. It is a slowly progressive lung disease, characterized by chronic airway inflammation and not fully reversible airflow obstruction [3]. Cigarette smoking is the main cause of COPD in developed countries, and patients typically present with shortness of breath, chronic cough and/or excessive sputum production. In addition to a higher risk of mortality and morbidity, COPD is associated with a substantial economic burden of illness to the health care system [4-6].

The management of COPD is largely symptomatic, so patient-reported outcomes (PROs) that evaluate health-related quality of life (HRQL) are important to evaluating the treatment and management of COPD. As COPD progresses, poor symptom control and exacerbations can lead to limitations in functioning and impaired HRQL [7]. Disease-specific measures can provide insight into specific aspects of HRQL, while generic HRQL measures have the advantage of being able to compare across different patient populations but they may be less sensitive to changes in HRQL compared to disease-specific measures [8,9].

Two generic HRQL measures, the Patient Reported Outcomes Measurement Information System (PROMIS) and EQ-5D-5L, were recently developed that have potential for broad use in evaluating COPD outcomes. PROMIS is a health measurement system designed for a wide variety of patient populations that utilizes banks of items belonging to specific domains of health [10]. The PROMIS item banks were derived using item response theory (IRT) and developed through a rigorous process of literature review, focus groups across multiple diseases and sites, cognitive assessments, and expert consultation. In addition, fixed-length short forms, including the PROMIS-43, were developed as an alternative computer-adaptive testing (CAT). These short forms, while not providing as precise measurement estimates as using CAT, cover core dimensions of health from the PROMIS. Another measure, the EQ-5D-5L, expanded the EQ-5D from 3 Levels to 5 Levels in order to potentially improve upon the properties of the standard 3-level EQ-5D by enhancing sensitivity and reducing ceiling effects [11].

As few studies have examined these recently developed measures in COPD, the aims of this study were: (1) to examine their psychometric properties in patients with COPD, and (2) to identify dimensions of HRQL that differ and do not differ by lung function.

## Methods

### Study design and subject recruitment

We conducted a secondary data analysis on COPD patients who participated in a multi-center cross-sectional study (NHLBI COPD Outcomes-based Network for Clinical Effectiveness & Research Translation [CONCERT], https://www.kpchr.org/concert/). The CONCERT investigators developed a COPD Data Warehouse (CDW), containing comprehensive information on more than 220,000 patients with some indication of a chronic respiratory condition between 2006 and 2010. Seven U.S. clinical centers were involved in patient recruitment: Kaiser Permanente Northwest Region, VA Puget Sound Health Care System, University of Chicago, University of Illinois Hospital and Health Sciences System, University of Washington, Baystate Medical Center, and University of North Carolina at Chapel Hill. Institutional Review Board approval was received at each individual site and at the Data Coordinating Center. A sample of patients from the CDW were recruited for in-person evaluations and a total of 1,206 patients (response rate 36%) participated and completed the evaluations including spirometry, six-minute walk test (6MWT), and extensive patient-reported outcomes (dyspnea scores, quality of life, etc) (see Additional file 1 for details on patient sampling and recruitment). Patients were excluded if they could not perform a spirometry test or could not participate due to cognitive impairment, frailty, acute illness, receiving hospice care or staying in long-term care facility, and issues related to geography, administration or communication.

For the present study, patients fulfilling all the following criteria were included: (1) having a diagnosis of COPD, defined based on the Global Initiative for Chronic Obstructive Lung Disease (GOLD) spirometric criteria—a post-bronchodilator FEV_1_/FVC (forced expiratory volume in 1 second/forced vital capacity) ratio <0.7 [3]; (2) ≥40 years of age (as COPD mainly affects people over the age of 40 and the disease develops over decades of exposure to inhaled particulates [12]); (3) with FEV_1_ percent predicted available to indicate COPD disease severity; and (4) completed both EQ-5D-5L and PROMIS questionnaires.

### Measures

Clinical and HRQL measures were administered to every patient. Post-bronchodilator spirometry was utilized to assess the extent of airflow limitation. Severity of COPD was graded using percent predicted FEV_1_: GOLD 1 (mild) – FEV_1_ ≥ 80% predicted, GOLD 2 (moderate) – 50% ≤ FEV_1_ < 80% predicted, GOLD 3 (severe) – 30% ≤ FEV_1_ < 50% predicted, and GOLD 4 (very severe) – FEV_1_ < 30% predicted [3].

The six minute walk test (6MWT) was administered to assess patients’ functional capacity. The 6MWT measures the distance (in meters) that an individual is able to walk on a flat and hard floor over six minutes, referred to as the 6 minute walk distance (6MWD) [13]. Dyspnea scales were used to quantify the degree of shortness of breath. These included the modified Medical Research Council (mMRC) dyspnea scale, Borg scale, and the Functional Assessment of Chronic Illness Therapy (FACIT)-Dyspnea 10-item short form. A higher score is associated with more severe symptoms. The widely used mMRC dyspnea scale is easy to administer, where patients indicate their dyspnea level on a 5-point scale (0-4) by selecting the physical tasks that provoke shortness of breath [14]. Borg scale is another method of rating breathlessness, both at rest and during the 6MWT (measuring exertional dyspnea), on a scale of 0 to 10 [15]. The FACIT-Dyspnea short form comprises 10 items that describe the experience of shortness of breath when doing a range of common tasks in the past week, with each item scored on a 4-point rating scale (from 0 [no shortness of breath] to 3 [severe shortness of breath]) [16,17]. FACIT-Dyspnea scores are generated using the *T*-score metric, where summary scores from responses to items are transformed into a scale with a mean of 50 and a standard deviation [SD] of 10 for patients with self-reported COPD [18].

EQ-5D is a widely used preference-based measure that includes a descriptive health self-classifier and a visual analog scale (EQ-VAS) [19]. The classifier contains five dimensions of health: mobility, self-care, usual activities, pain/discomfort and anxiety/depression. The original EQ-5D, which has 3 Levels (EQ-5D-3L) was recently expanded to 5 Levels (EQ-5D-5L) for each single item dimension of health. An index-based summary score for the EQ-5D-5L can be generated using a recently published crosswalk between the EQ-5D-5L descriptive system and the EQ-5D-3L value sets [20]. This algorithm provides index-based scores ranging from -0.109 to 1.0 in the U.S. population, with lower values signifying worse health [21]. The EQ-VAS asks the patient to rate their own health on a scale ranging from 0 (worst imaginable health) to 100 (best imaginable health) [19].

The PROMIS-43 short form, a multi-dimensional 43-item generic measure of health, is intended for use across a variety of conditions. It includes seven domains: physical function, anxiety, depression, fatigue, sleep disturbance, pain interference, and satisfaction with participation in social roles, as well as a pain intensity item scored from 0 to 10. There are 6 items in each of the seven domains, with responses ranging from 1 to 5. Raw scores for each domain are calculated by summing the item scores while adjusting for missing item responses, and it can be estimated if at least 4 out of the 6 items in that domain were answered. Raw scores are transformed using the *T*-score metric based on the item response theory calibrations in which scores have a mean of 50 and a SD of 10 for the general population in the U.S. [22,23]. T-scores can be estimated using the scoring tables listed in the PROMIS manuals [24]. A higher PROMIS T-score implies more of the concept being measured; for instance, a higher PROMIS score on physical function indicates better functioning, whereas a higher score on depression indicates more severe depressive symptoms.

### Statistical analysis

The psychometric properties of EQ-5D-5L and PROMIS-43 were examined in terms of reliability, convergent validity, and discriminative ability. Internal consistency reliability of the PROMIS domains was evaluated using Cronbach’s alpha. The distribution of responses to the EQ-5D-5L were described, and Chi-square or Fisher’s exact tests were performed to assess the ability of EQ-5D dimensions to discern among COPD patients with different grades of airflow limitation.

Convergent construct validity was evaluated by assessing the strength of associations among the PROMIS domains, EQ-5D measures (summary and dimension scores), and other clinical or functional status measures using Spearman’s rank correlation coefficients (r_s_). Correlation coefficients were interpreted as follows: r_s_ < 0.10 (absent), r_s_ = 0.10-0.29 (weak), r_s_ = 0.30-0.49 (moderate), and r_s_ ≥ 0.50 (strong) [25]. We hypothesized that strong correlations would be observed between related domains of health on the EQ-5D-5L and PROMIS, i.e. mobility and physical functioning; usual activities and social roles; pain/discomfort and pain interference; anxiety/depression and anxiety and depression, respectively.

One-way analysis of variance (ANOVA) was used to compare the EQ-5D, EQ-VAS, PROMIS domains, and other measures among patients with different GOLD grades. ANOVA F-statistics comparing mean scores across GOLD grades were used to assess the relative discriminative ability of the EQ summary scores (index score, VAS) and the PROMIS-43 subscales. Relative statistical efficiency (RE) was compared, defined as the ratio of the ANOVA F-statistics for a given measure and the FACIT-Dyspnea score, which served as the reference measure [26-28]. Although ANOVA is robust to moderate deviations from normality [29], non-parametric Kruskal-Wallis tests were also performed. All statistical analyses were carried out using the Statistical Analysis System (SAS), version 9.2 (SAS Institute, Cary, NC). All p-values were reported, and for purposes of interpretation, p-values <0.01 were considered statistically significant.

## Results

A total of 670 patients were included in our analysis. The mean age was 68.5 (SD 10.4) years, with 58% men and 78% self-identified as caucasian (Table 1). Most patients were current (n = 193, 28.8%) or past (n = 412, 61.5%) smokers, with an average of 44.2 (SD 31.2) pack-years. The study subjects were divided into 4 groups based on the severity of airflow limitation: mild (GOLD 1, n = 102), moderate (GOLD 2, n = 353), severe (GOLD 3, n = 165) and very severe (GOLD 4, n = 50). Patients included as GOLD 3 and 4 (with more severe disease) were significantly younger, more likely to be African-American, have lower education level, less household income, heavier smoking history, and less likely to have coronary heart disease as compared to those with mild to moderate disease (p < 0.05) (Table 1).

**Table 1:** Patient demographic and clinical characteristics (Total N = 670)

**Characteristic, n (%)**	**No. of missing**	**Total sample**	**GOLD 1 (Mild; n = 102)**	**GOLD 2 (Moderate; n = 353)**	**GOLD 3 (Severe; n = 165)**	**GOLD 4 (Very severe; n = 50)**	**p-value (comparing GOLD 1,2 vs 3,4)**
Age, mean (SD)	0	68.5 (10.4)	72.1 (11.3)	68.3 (10.5)	67.7 (9.7)	65.1 (7.4)	0.02^§^
Male	0	387 (57.8)	69 (67.7)	203 (57.5)	88 (53.3)	27 (54.0)	0.12^†^
Race	0						<.0001^‡^
Caucasian		524 (78.2)	91 (89.2)	290 (82.2)	114 (69.3)	29 (58.0)	
African American		121 (18.1)	11 (10.8)	52 (14.7)	40 (24.1)	18 (36.0)	
Native American*		20 (3.0)	0 (0)	8 (2.3)	9 (5.4)	3 (6.0)	
Asian/multiracial/other		5 (0.8)	0 (0)	3 (0.9)	2 (1.2)	0 (0)	
BMI (Kg/m^2^), mean (SD)	4	29.1 (7.6)	29.1 (6.2)	29.4 (7.0)	29.4 (8.9)	26.0 (9.3)	0.26^§^
Main activity	0						0.30^†^
Employed (incl.self-employment)		106 (15.8)	18 (17.7)	61 (17.3)	20 (12.1)	7 (14.0)	
Retired		451 (67.3)	69 (67.7)	234 (66.3)	114 (69.1)	34 (68.0)	
Keeping house/student/seeking work		61 (9.1)	10 (9.9)	31 (8.8)	17 (10.3)	3 (6.0)	
Disabled – on disability		41 (6.1)	5 (4.9)	22 (6.2)	8 (4.9)	6 (12.0)	
Other		11 (1.6)	0 (0)	5 (1.4)	6 (3.6)	0 (0)	
Education	0						0.04^†^
Some high school or less		92 (13.7)	12 (11.8)	48 (13.6)	27 (16.4)	5 (10.0)	
High school graduate or GED		178 (26.6)	23 (22.6)	97 (27.5)	44 (26.7)	14 (28.0)	
Vocational college or some college		256 (38.2)	36 (35.3)	126 (35.7)	70 (42.4)	24 (48.0)	
College degree		86 (12.8)	14 (13.7)	53 (15.0)	16 (9.7)	3 (6.0)	
Professional or graduate degree		58 (8.7)	17 (16.7)	29 (8.2)	8 (4.9)	4 (8.0)	
Household total gross yearly income	77						0.002^†^
Less than $30,000		275 (46.4)	30 (36.1)	137 (43.5)	86 (57.3)	22 (48.9)	
$30,001 to $50,000		164 (27.7)	25 (30.1)	94 (29.8)	33 (22.0)	12 (26.7)	
$50,001 to $75,000		82 (13.8)	14 (16.9)	39 (12.4)	23 (15.3)	6 (13.3)	
Over $75,000		72 (12.1)	14 (16.9)	45 (14.3)	8 (5.3)	5 (11.1)	
Smoking status	0						0.09^†^
Current smoker		193 (28.8)	21 (20.6)	109 (30.9)	49 (29.7)	14 (28.0)	
Past smoker		412 (61.5)	64 (62.8)	209 (59.2)	104 (63.0)	35 (70.0)	
Never smoker		65 (9.7)	17 (16.7)	35 (9.9)	12 (7.3)	1 (2.0)	
Smoke pack-years, mean (SD)	2	44.2 (31.2)	36.3 (30.3)	44.2 (30.9)	48.0 (31.5)	48.2 (31.8)	0.03^§^
Comorbid medical conditions							
Coronary heart disease**	0	220 (32.8)	42 (41.2)	119 (33.7)	51 (30.9)	8 (1.2)	0.04^†^
Hypertension	0	416 (62.1)	57 (55.9)	221 (62.6)	110 (66.7)	28 (56.0)	0.44^†^
Diabetes	0	154 (23.0)	20 (19.6)	85 (24.1)	39 (23.6)	10 (20.0)	0.93^†^
Stroke or cerebrovascular disease	0	96 (14.3)	13 (12.8)	53 (15.0)	25 (15.2)	5 (10.0)	0.85^†^
Cancer history	0	174 (26.0)	22 (21.6)	96 (27.2)	44 (26.7)	12 (24.0)	0.98^†^
Dementia	0	10 (1.5)	2 (2.0)	7 (2.0)	1 (0.6)	0 (0)	0.18^‡^
Depression	0	229 (34.2)	34 (33.3)	131 (37.1)	52 (31.5)	12 (24.0)	0.10^†^

GED = general education diploma.

Based on ^†^Chi-square, or ^‡^Fisher’s exact test or ^§^t-test.

*Inclde American Indian, Alaskan Native, Native Hawaiian, Pacific Islander, Native American plus other.

**Include angina, coronary artery disease, coronary artery revascularization, or myocardial infarction diagnosed by doctor.

All levels of the EQ-5D-5L were utilized by the overall cohort, but only a relatively small proportion of patients reported severe or extreme problems on each dimension (range 1.4-8.7%) (Table 2). More than 50% of patients reported no problems with self-care and anxiety/depression (in all COPD grades) and usual activities (GOLD 1 only). More severe COPD was associated with significantly more problems with mobility, self-care and usual activities (p < 0.01). Approximately 30% of all COPD patients reported at least moderate pain/discomfort and 15% reported at least moderate anxiety/depression, but differences across COPD grades were not significant (p = 0.26 and p = 0.15, respectively). The response of ‘11111’ (no problems in any dimension) was reported in a dimishing proportion of respondents as grades got more severe: 19.6% (GOLD 1), 18.4% (GOLD 2), 11.5% (GOLD 3), and 4% (GOLD 4). Overall, the mean (SD) score was 0.79 (0.15) for the EQ-5D index, and 70.6 (19.6) for the EQ-VAS (Table 3). When stratified by GOLD grade, EQ-5D index-based mean scores ranged from 0.81 (GOLD 1) to 0.74 (GOLD 4) (p-value = 0.004), and EQ-VAS mean scores ranged from 76.6 (GOLD 1) to 61.1 (GOLD 4) (p-value < 0.001). Patients with more severe COPD had lower mean EQ-5D index scores and EQ-VAS scores, although the index-based score did not discriminate between the milder grades of COPD.

**Table 2:** EQ-5D-5L profile of patients by GOLD grade

	**Total sample [n (%)]**	**GOLD 1 (mild; n = 102) [n (%)]**	**GOLD 2 (moderate; n = 353) [n (%)]**	**GOLD 3 (severe; n = 165) [n (%)]**	**GOLD 4 (very severe; n = 50) [n (%)]**	**p-value (comparing GOLD 1,2 vs 3,4)**
EQ-5D dimension						
Mobility						0.003^†^
No problems	244 (36.4)	44 (43.1)	141 (39.9)	41 (24.9)	18 (36.0)	
Slight problems	179 (26.7)	28 (27.5)	93 (26.4)	46 (27.9)	12 (24.0)	
Moderate problems	189 (28.2)	25 (24.5)	92 (26.1)	58 (35.2)	14 (28.0)	
Severe problems	52 (7.8)	5 (4.9)	25 (7.1)	16 (9.7)	6 (12.0)	
Extreme problems	6 (0.9)	0 (0)	2 (0.6)	4 (2.4)	0 (0)	
Self-care						<.0001^‡^
No problems	539 (80.5)	87 (85.3)	297 (84.1)	127 (77.0)	28 (56.0)	
Slight problems	88 (13.1)	13 (12.8)	43 (12.2)	23 (13.9)	9 (18.0)	
Moderate problems	34 (5.1)	2 (2.0)	9 (2.6)	11 (6.7)	12 (24.0)	
Severe problems	5 (0.8)	0 (0)	4 (1.1)	0 (0)	1 (2.0)	
Extreme problems	4 (0.6)	0 (0)	0 (0)	4 (2.4)	0 (0)	
Usual activities						<.0001^†^
No problems	303 (45.2)	61 (59.8)	175 (49.6)	56 (33.9)	11 (22.0)	
Slight problems	183 (27.3)	22 (21.6)	96 (27.2)	49 (29.7)	16 (32.0)	
Moderate problems	135 (20.2)	13 (12.8)	64 (18.1)	44 (26.7)	14 (28.0)	
Severe problems	32 (4.8)	4 (3.9)	12 (3.4)	10 (6.1)	6 (12.0)	
Extreme problems	17 (2.5)	2 (2.0)	6 (1.7)	6 (3.6)	3 (6.0)	
Pain/discomfort						0.26^†^
No problems	255 (38.1)	35 (34.3)	131 (37.1)	62 (37.6)	27 (54.0)	
Slight problems	207 (30.9)	33 (32.4)	118 (33.4)	48 (29.1)	8 (16.0)	
Moderate problems	160 (23.9)	27 (26.5)	81 (23.0)	42 (25.5)	10 (20.0)	
Severe problems	43 (6.4)	7 (6.9)	21 (6.0)	11 (6.7)	4 (8.0)	
Extreme problems	5 (0.8)	0 (0)	2 (0.6)	2 (1.2)	1 (2.0)	
Anxiety/depression						0.15^†^
No problems	427 (63.7)	63 (61.8)	237 (67.1)	101 (61.2)	26 (52.0)	
Slight problems	139 (20.8)	20 (19.6)	64 (18.1)	41 (24.9)	14 (28.0)	
Moderate problems	79 (11.8)	16 (15.7)	40 (11.3)	15 (9.1)	8 (16.0)	
Severe problems	20 (3.0)	2 (2.0)	11 (3.1)	5 (3.0)	2 (4.0)	
Extreme problems	5 (0.8)	1 (1.0)	1 (0.3)	3 (1.8)	0 (0)	

GOLD = Global initiative for chronic Obstructive Lung Disease.

Based on ^†^Chi-square, or ^‡^Fisher’s exact test.

**Table 3:** Patient clinical and HRQL measurements

**Measure**	**No. of missing**	**Total sample [Mean (SD)]**	**GOLD 1 (n = 102) [Mean (SD)]**	**GOLD 2 (n = 353) [Mean (SD)]**	**GOLD 3 (n = 165) [Mean (SD)]**	**GOLD 4 (n = 50) [Mean (SD)]**	**ANOVA**	**RE ratio**	**ANOVA**	**Kruskal-Wallis test**
							**F-stat**		**p-value**	**p-value**
FACIT-Dyspnea	6	44.6 (8.4)	41.0 (7.9)	43.3 (8.0)	47.6 (8.0)	51.6 (6.9)	31.19	Ref	<.0001	<.0001
mMRC dyspnea	60	1.50 (0.99)	1.20 (0.95)	1.38 (0.95)	1.79 (0.95)	1.88 (1.06)	11.71	0.38	<.0001	<.0001
Borg dyspnea (at rest)	55	0.67 (1.08)	0.55 (0.91)	0.60 (1.07)	0.92 (1.19)	0.64 (0.97)	3.52	0.11	0.01	0.02
Borg dyspnea (during 6MWT)	60	2.52 (1.94)	2.07 (1.73)	2.27 (1.90)	3.02 (1.78)	3.78 (2.37)	13.06	0.42	<.0001	<.0001
6MWD	60	335.6 (110.4)	371.9 (111.2)	345.5 (109.4)	301.2 (103.1)	297.8 (101.3)	11.24	0.36	<.0001	<.0001
EQ-5D index	0	0.79 (0.15)	0.81 (0.14)	0.81 (0.14)	0.76 (0.17)	0.74 (0.15)	6.13	0.20	0.0004	0.002
EQ-VAS	0	70.6 (19.6)	76.6 (17.5)	72.6 (19.1)	65.7 (20.2)	61.1 (19.7)	12.24	0.39	<.0001	<.0001
PROMIS										
Physical function (P-PF)	0	40.6 (7.6)	43.2 (8.2)	41.7 (7.7)	38.3 (6.0)	35.3 (5.5)	21.41	0.69	<.0001	<.0001
Anxiety (P-A)	0	49.7 (9.2)	49.5 (9.6)	49.3 (9.0)	50.0 (9.4)	52.3 (8.7)	1.69	0.05	0.17	0.15
Depression (P-D)	0	48.3 (9.3)	49.3 (10.1)	47.3 (8.8)	48.5 (9.6)	51.8 (8.9)	4.08	0.13	0.007	0.01
Fatigue (P-F)	0	50.8 (9.2)	51.2 (9.9)	50.0 (9.2)	51.3 (8.8)	53.9 (8.4)	3.01	0.10	0.03	0.04
Sleep disturbance (P-SD)	0	49.8 (9.4)	51.5 (10.2)	49.3 (9.0)	50.0 (9.6)	48.9 (9.4)	1.64	0.05	0.18	0.23
Social roles (P-SR)	2	48.1 (9.3)	49.3 (9.0)	49.0 (9.4)	46.3 (9.3)	44.7 (7.9)	6.07	0.19	0.0004	0.001
Pain interference (P-PI)	3	53.9 (9.6)	53.8 (9.5)	54.3 (9.5)	53.3 (9.7)	53.1 (9.8)	0.56	0.02	0.64	0.74
Pain intensity scale (P-P)	0	3.49 (2.67)	3.49 (2.69)	3.54 (2.62)	3.43 (2.70)	3.28 (2.90)	0.18	0.01	0.91	0.85

6MWD = six-minute walk distance; 6MWT = six-minute walk test; ANOVA = analysis of variance; GOLD = Global initiative for chronic Obstructive Lung Disease; FACIT = Factional Assessment of Chronic Illness Therapy; mMRC = modified Medical Research Council; PROMIS = Patient Reported Outcomes Measurement Information System; RE = relative efficiency; ref = reference group (RE = 1).

Regarding PROMIS-43, physical function had a overall mean score of 40.6 (SD 7.6), and for the rest of domains, the mean scores ranged from 48.1 (social roles) to 53.9 (pain interference) (Table 3). The mean pain intensity score was 3.49 (SD 2.67) on a scale of 0 to 10. The PROMIS physical function, depression, fatigue, and social roles had p-values <0.05, but only physical function and social roles demonstrated a statistically significant decline that was monotonically associated with decreasing lung function (Table 3). No differences in mean scores by GOLD grade were observed for the PROMIS domains of anxiety, sleep disturbance, pain interference and pain intensity.

About sixty patients refused or did not complete the 6MWT and/or dyspnea severity assessment due to health issues (e.g., wheelchair or walker dependent, body pain, discomfort while doing the test). All the dyspnea measures and 6MWD demonstrated discriminative ability when mean scores were compared among subgroups with different levels of COPD severity (Table 3). The FACIT-Dyspnea provided the highest relative efficiency (RE) to discriminate among subgroups of COPD severity, followed by PROMIS physical function, Borg dyspnea during 6MWT, EQ-VAS, mMRC dyspnea, 6MWD, and EQ-5D index .

Cronbach’s alpha for PROMIS domains ranged from 0.89 to 0.95, demonstrating acceptable internal consistency reliability [30]. Strong correlations between related domains on the EQ-5D-5L and PROMIS-43 were observed as expected (Table 4). EQ-5D usual activities (EQ-UA) showed strong correlations with the PROMIS physical function (P-PF) (*r*_*s*_ = -0.65), fatigue (P-F) (*r*_*s*_ = 0.54), and social roles (P-SR) (*r*_*s*_ = -0.55), and moderate correlations with the rest of PROMIS domains. EQ-5D pain/discomfort (EQ-PD) was strongly correlated with PROMIS pain interference (P-PI) and pain intensity (P-P) (*r*_*s*_ = 0.67 and 0.63, respectively), and the EQ-5D anxiety/depression (EQ-AD) with PROMIS anxiety (P-A) and depression (P-D) (*r*_*s*_ = 0.60 and 0.59, respectively). EQ-5D mobility (EQ-MO) was moderately to strongly related to four domains of the PROMIS (physical function [P-PF], fatigue [P-F], social roles [P-SR], and pain interference [P-PI]) and the pain intensity item (P-P). The EQ-5D dimension of self-care (EQ-SC) was moderately correlated with P-PF and P-SR.

**Table 4:** **Correlations between domains of EQ-5D-5L and PROMIS-43 (all with *p *** **<0.001)**

	**EQ-MO**	**EQ-SC**	**EQ-UA**	**EQ-PD**	**EQ-AD**	**P-PF**	**P-A**	**P-D**	**P-F**	**P-SD**	**P-SR**	**P-PI**
EQ-MO	1											
EQ-SC	0.39	1										
EQ-UA	0.52	0.45	1									
EQ-PD	0.47	0.23	0.38	1								
EQ-AD	0.26	0.22	0.38	0.34	1							
P-PF	-0.65	-0.46	-0.65	-0.40	-0.34	1						
P-A	0.23	0.21	0.34	0.29	0.60	-0.40	1					
P-D	0.28	0.26	0.37	0.30	0.59	-0.44	0.78	1				
P-F	0.41	0.27	0.54	0.40	0.41	-0.62	0.53	0.59	1			
P-SD	0.20	0.15	0.34	0.28	0.32	-0.34	0.41	0.45	0.55	1		
P-SR	-0.44	-0.34	-0.55	-0.32	-0.37	0.65	-0.45	-0.49	0.61	-0.42	1	
P-PI	0.46	0.23	0.44	0.67	0.35	-0.52	0.42	0.43	0.57	0.41	-0.47	1
P-P	0.40	0.19	0.34	0.63	0.32	-0.43	0.39	0.38	0.45	0.41	-0.40	0.79

EQ-MO = EQ-Mobility; EQ-SC = EQ-Self-Care; EQ-UA = EQ-Usual Activities; EQ-PD = EQ-Pain/Discomfort; EQ-AD = EQ-Anxiety/Depression; PROMIS = Patient Reported Outcomes Measurement Information System; P-PF = PROMIS-Physical Function; P-A = PROMIS-Anxiety; P-D = PROMIS-Depression; P-F = PROMIS-Fatigue; P-SD = PROMIS-Sleep Disturbance; P-PI = PROMIS-Pain Interference; P-P = PROMIS-Pain intensity scale; P-SR = PROMIS-Satisfaction with participation in Social Roles.

In examining the relationship between clinical measures and the EQ-5D index, EQ-VAS, PROMIS subscales, only the FACIT-Dyspnea and the PROMIS physical function (P-PF) showed at least moderate correlations with % of predicted FEV_1_ (*r*_*s*_ *=* -0.36 and 0.32, respectively) (Table 5). EQ-5D index scores and VAS scores were both moderately to strongly correlated with PROMIS domains, dyspnea scales, and the 6MWD, but the magnitude of these correlations was smaller with EQ-VAS scores than EQ-5D index scores. All subscales of PROMIS-43 had moderate to strong correlations with at least one of the dyspnea scores. Among all the HRQL measures, the PROMIS physical function (P-PF), fatigue (P-F), social roles (P-SR), EQ-5D index and EQ-VAS, in general, had stronger correlations with the symptom severity. All measures were at least (or nearly) moderately correlated with 6MWD, except for PROMIS anxiety (P-A), depression (P-D) and sleep disturbance (P-SD) (absolute *r* <0.3).

**Table 5:** Correlations between clinical and HRQL measures

	**% pred. FEV**_**1**_	**FACIT- Dyspnea**	**mMRC dyspnea**	**Borg dyspnea (at rest)**	**Borg dyspnea (during 6MWT)**	**6MWD**	**EQ-5D index**	**EQ-VAS**
% pred. FEV_1_	1							
FACIT-dyspnea	-0.36	1						
mMRC dyspnea	-0.29	0.59	1					
Borg dyspnea (at rest)	-0.13	0.46	0.33	1				
Borg dyspnea (during 6MWT)	-0.26	0.56	0.48	0.36	1			
6MWD	0.28	-0.44	-0.45	-0.25	-0.34	1		
EQ-5D index	0.19	-0.58	-0.48	-0.38	-0.37	0.46	1	
EQ-VAS	0.26	-0.43	-0.41	-0.34	-0.35	0.29	0.55	1
P-PF	0.32	-0.71	-0.62	-0.39	-0.58	0.56	0.68	0.51
P-A	-0.09	0.42	0.23	0.27	0.23	-0.19	-0.46	-0.33
P-D	-0.08	0.45	0.25	0.26	0.29	-0.19	-0.49	-0.34
P-F	-0.09	0.58	0.43	0.38	0.42	-0.29	-0.55	-0.48
P-SD	0.02^†^	0.35	0.28	0.26	0.23	-0.10	-0.37	-0.31
P-SR	0.18	-0.57	-0.41	-0.37	-0.42	0.37	0.54	0.47
P-PI	-0.01^†^	0.41	0.33	0.31	0.22	-0.29	-0.65	-0.37
P-P	0.01^†^	0.35	0.26	0.27	0.21	-0.29	-0.58	-0.33

6MWD = six-minute walk distance; 6MWT = six-minute walk test; FACIT = Factional Assessment of Chronic Illness Therapy; FEV_1_ = forced expiratory volume in 1 second; mMRC = modified Medical Research Council; PROMIS = Patient Reported Outcomes Measurement Information System; P-PF = PROMIS-Physical Function; P-A = PROMIS-Anxiety; P-D = PROMIS-Depression; P-F = PROMIS-Fatigue; P-SD = PROMIS-Sleep Disturbance; P-PI = PROMIS-Pain Interference; P-P = PROMIS-Pain intensity scale; P-SR = PROMIS-Satisfaction with participation in Social Roles.

^†^Spearman’s rank correlation coefficient *p >* 0.05 (not significant).

## Discussion

Our study results provide evidence to support the validity of two recently developed generic measures of HRQL, EQ-5D-5L and PROMIS-43, in COPD. The convergent construct validity of the two measures was supported by the moderate to strong correlations between related domains, and between the domain and summary scores of the generic measures and the dyspnea measures. EQ-5D-5L index, EQ-VAS, and two domains of PROMIS (physical function and social roles) had higher RE ratios among the HRQL measures, suggesting that these scores provide greater statistical power (discriminative ability) to capture differences in HRQL in relation to disease severity as measured by lung function.

Level of dyspnea is a strong predictor for health status [31-33]. Both EQ-5D and PROMIS had moderate associations with at least one measure of dyspnea, with the correlations varying across the PROMIS-43 subscales. Our results concur with previous reports that spirometric parameters (% of predicted FEV_1_), unlike severity of breathlessness, does not correlate well with HRQL [31,32,34,35]. While lung function test with spirometry serves as an important clinical tool to measure the degree of airflow limitation, a number of studies have demonstrated that it provides an incomplete assessment of health burden to the patient, which can include physical and psychosocial functioning. This discernment coincides with the new COPD assessment tool recently proposed by the GOLD, which recommends evaluation of COPD based on not only lung function, but also the assessment of symptoms and exacerbation risk [3]. This also reinforces the importance of evaluating patient-reported outcomes along with clinical measures (e.g., lung function test) when gauging the effect of health interventions.

COPD severity has been shown to influence the degree of physical disability, impairing the ability to perform activities of daily living, and contributing to poor HRQL [36]. Patients with COPD had relatively worse self-rated HRQL in multiple PROMIS domains as compared with individuals without COPD or any condition [37]. The negative impact of COPD is more pronounced on the physical aspect of health than on the mental component [31]. Consistent with the study by Gonzalez-Moro and colleagues [36], our findings suggest that, in general, physical functioning tends to be affected in all grades of COPD patients while mental health is impaired only in patients at more severe stages. The mean scores of PROMIS domains indicated that physical function, among all the domains measured in the PROMIS, was the aspect of health status most affected by COPD; physical function was considerably impaired even in patients with mild COPD, as the mean domain score of PROMIS physical function in patients with GOLD grade 1 was less than 50 (the mean score of the general population). The mean domain scores of PROMIS anxiety and depression were higher than 50 only in patients with very severe COPD (i.e. GOLD 4).

Evidence on the properties of the EQ-5D-5L is only beginning to emerge. The first paper was a multi-country study by Janssen et al. in 2012 that compared the measurement properties of the 5-level and 3-level EQ-5D, including 342 patients with respiratory disease (COPD or asthma) as one of the eight patient groups with chronic conditions [38]. The 5-level EQ-5D descriptive system (EQ-5D-5L) reduced ceiling effects of the 3-level EQ-5D (EQ-5D-3L) and improved the discriminatory power and convergent validity. In our study, broad use of 4 of the 5 Levels of the EQ-5D-5L suggests that it could provide higher discriminative power than the standard EQ-5D-3L in COPD, although the most severe category appears to be rarely utilized. A previous study showed that EQ-5D-3L index score (both UK and US) failed to differ across COPD severity stages [35]. The mean EQ-5D-5L index score significantly decreases as COPD severity deteriorates, particularly in the advanced stages of disease (Table 3), which may suggest better discriminatory power of EQ-5D-5L than EQ-5D-3L to distinguish COPD patients of different severity. Similar to studies of the EQ-5D-3L in COPD, self-care is the dimension least affected by COPD [39,40]. In accordance with a study by Punekar et al. [40], about 80% of COPD patients reported no problems in self-care. As the severity of COPD increased, COPD patients reported more problems with mobility, self-care, and usual activities. However, pain/discomfort and anxiety/depression tended not to differ by disease severity using the EQ-5D-5L or the PROMIS. Our study also suggested that EQ-5D-5L index scores were less able to discriminate among patients with milder disease, i.e. GOLD grades 1 and 2. This is consistent with a previous study by Antonelli-Incalzi et al. who observed that health status dramatically declined when predicted FEV_1_ was 49% or less (upper limit of GOLD grade 3) [41]. Alternatively, the lack of discrimination between grade 1 and 2 may also suggest that the EQ-5D-5L descriptive system does not entirely address some of the limitations of the three-level EQ-5D [39], assuming there is a meaningful difference in self-reported health based on GOLD grades 1 and 2. Unlike the EQ-5D index score which is derived based on the five dimensions using population preference weights, the EQ-VAS provides a direct rating of health from the patient’s point of view. Consistent with previous reports [35], EQ-VAS had a more monotonic relationship with disease severity and better ability to discriminate according to disease severity compared to EQ-5D index.

Among the PROMIS subscales, physical functioning was most strongly associated with disease grades and measures of breathing difficulty and functioning. Only physical function (P-PF), depression (P-D), fatigue (P-F), and social roles (P-SR) varied significantly across COPD grades and the magnitude of differences in the PROMIS scores of depression and fatigue across different GOLD grades were smaller than half of their SD, a commonly used cutoff for interpreting important differences in HRQL scores [42]. Anxiety, sleep, and pain domains of PROMIS, although moderately related to other HRQL measurements and dyspnea scores (mainly FACIT-Dyspnea), did not vary by COPD GOLD grade. The lack of correlation between pain, anxiety, and sleep disturbance and the degree of COPD severity does not preclude the importance of these HRQL parameters in COPD patients. In fact, it has been reported that 35%, 37% and 51% of advanced COPD patients suffer from sleep disturbance, pain and anxiety, respectively, arguably among the most prevalent symptoms associated with advanced COPD [43]. Despite the inability of these domains to discriminate patients with different level of airflow limitation, the domains present convergent validity and it suggests that they may capture patient-reported outcomes other than those associated with spirometry. In addition, the observation that the parameters of physical or physiological measures (dyspnea scores; mobility, self-care and usual activities in EQ-5D-5L; physical function in PROMIS) deteriorate more with the increase in COPD severity, as compared to psychosocial measures (anxiety/depression in EQ-5D-5L; anxiety, depression and social roles in PROMIS), suggests the possibility of adapation and coping mechanisms developed in COPD patients as the disease severity progresses, which is often observed with chronic illnesses and disabling conditions [44].

EQ-5D and PROMIS, both generic measures of HRQL, are distinctive in several ways. While EQ-5D index and VAS scores both provide summary scores for evaluating general health status as a whole, PROMIS describes different aspect of health status individually using domain scores. The domains of anxiety, depression, and pain are apparently covered by both of the measures, but it is arguable if fatigue (PROMIS) overlaps with pain/discomfort (EQ-5D), as well as the extent of overlap between physical functioning, fatigue, sleep disturbance, or social roles (PROMIS) and mobility, self-care, or usual activities (EQ-5D). EQ-5D index-based scores are generated from societal preferences for health that can be applied to economic evaluations. Although PROMIS-43 does not include global items and was not designed as a preference-based measure as EQ-5D, at least one scoring function is available to convert PROMIS selected domain scores into a single index value by mapping onto the EQ-5D [45]. Comparing to PROMIS, EQ-5D is presumably briefer to administer as it contains 6 items (including VAS) rather than 43, but the PROMIS-43 contains more items in each domain, thereby providing the potential of a higher level of precision and sensitity than EQ-5D. Alternatively, even briefer short-form versions of the PROMIS are available.

This study has several limitations. Since patients did not complete EQ-5D-3L, we could not directly determine whether the EQ-5D-5L improves upon the properties of the EQ-5D-3L in COPD. In addition, longitudinal data are needed to examine and compare the responsiveness of the measures to detect meaningful change following interventions. Lastly, in our study, patients with more severe COPD (GOLD 3 and 4) were younger than those with milder disease, which was contrary to our expectation but may be due to the eligibility of study participation or possibly a survivor effect. The representativeness of patients included in this analysis could also be restricted by the relatively low response rate (36%) for participating in the in-person evaluations. Age is a known factor that could confound the association between HRQL and disease severity [7]. In order to rule out the confounding effect, we also conducted an analysis of covariance (ANCOVA) to control for age when comparing the responses in EQ-5D, PROMIS domain scores, dyspnea measures, and 6MWD among patients with different GOLD grades (data not shown). Similar results (F-statistic and significance level) were found as in Table 3 after controlling for age effect, except that the discriminative ability of 6MWD and PROMIS sleep disturbance (P-SD) to distinguish COPD patients of different severity was improved.

## Conclusions

In summary, our study provides evidence to support validity of EQ-5D-5L and PROMIS-43 to assess HRQL in patients with COPD. The measures indicated that patient-reported physical function and social activities decrease with level of lung function by GOLD grade, but not pain, mental health, sleep or fatigue. Future research using a longitudinal design will help to further understand the strengths and limitations of these measures in assessing outcomes in COPD patients.

## Abbreviations

6MWD: 6 minute walk distance; 6MWT: Six minute walk test; COPD: Chronic obstructive pulmonary disease; CONCERT: COPD Outcomes-based Network for Clinical Effectiveness & Research Translation; EQ-5D-5L: 5-level EuroQol-5 dimensions; EQ-VAS: EuroOol-Visual Analog Scale; FACIT-Dyspnea: Functional Assessment of Chronic Illness Therapy (FACIT)-Dyspnea 10-item short form; FEV_1_: Forced expiratory volume in 1 second; GOLD: Global Initiative for Chronic Obstructive Lung Disease; IRT: Item response theory; mMRC: Modified Medical Research Council; PROMIS: Patient Reported Outcomes Measurement Information System; RE: Relative efficiency.

## Competing interests

Dr. Simon Pickard is a member of the EuroQol group. No authors had financial conflicts of interest to disclose regarding the contents of this manuscript.

## Authors’ contributions

FJL participated in the study design, data analysis, data interpretation, and manuscript writing. ASP contributed to the supervision and design of the study, data interpretation, and revising the manuscript. TAL contributed in the study design, data collection, data interpretation, and manuscript revision. JAK, MJJ, DHA, SSC, SG, AGH, PKL, MAM, RAM, ETN, and WMV participated in the data collection and manuscript revision. All authors have read and approved the final manuscript.

## Pre-publication history

The pre-publication history for this paper can be accessed here:

http://www.biomedcentral.com/1471-2288/14/78/prepub

## Supplementary Material

Additional file 1 **Figure A1.** Flow diagram of CONCERT patient recruitment and follow-up. **Table A-1.** Pathways for inclusion in CONCERT-CER Data Warehouse. **Table A-2.** Definition of tiers used to define sampling strata. Click here for file

## References

[B1] Deaths: preliminary data for 2009 [http://www.cdc.gov/nchs/data/nvsr/nvsr59/nvsr59_04.pdf] 25073815

[B2] Mathers CD Loncar D Projections of global mortality and burden of disease from 2002 to 2030 PLoS Med 2006 3 11 e442 17132052 10.1371/journal.pmed.0030442 PMC1664601

[B3] Vestbo J Hurd SS Agusti AG Jones PW Vogelmeier C Anzueto A Barnes PJ Fabbri LM Martinez FJ Nishimura M Stockley RA Sin DD Rodriguez-Roisin R Global strategy for the diagnosis, management and prevention of chronic obstructive pulmonary disease: GOLD executive summary Am J Respir Crit Care Med 2013 187 4 347 365 22878278 10.1164/rccm.201204-0596PP

[B4] Morbidity and mortality: 2009 chartbook on cardiovascular, lung and blood diseases [http://www.nhlbi.nih.gov/resources/docs/2009_ChartBook.pdf]

[B5] Mapel DW Hurley JS Frost FJ Petersen HV Picchi MA Coultas DB Health care utilization in chronic obstructive pulmonary disease. A case-control study in a health maintenance organization Arch Intern Med 2000 160 17 2653 2658 10999980 10.1001/archinte.160.17.2653

[B6] Sullivan SD Ramsey SD Lee TA The economic burden of COPD Chest 2000 117 2 Suppl 5S 9S 10673466 10.1378/chest.117.2_suppl.5s

[B7] Stahl E Lindberg A Jansson SA Ronmark E Svensson K Andersson F Lofdahl CG Lundback B Health-related quality of life is related to COPD disease severity Health Qual Life Outcomes 2005 3 56 16153294 10.1186/1477-7525-3-56 PMC1215504

[B8] Kaplan RM Tally S Hays RD Feeny D Ganiats TG Palta M Fryback DG Five preference-based indexes in cataract and heart failure patients were not equally responsive to change J Clin Epidemiol 2011 64 5 497 506 20685077 10.1016/j.jclinepi.2010.04.010 PMC3973151

[B9] Patrick DL Deyo RA Generic and disease-specific measures in assessing health status and quality of life Med Care 1989 27 3 Suppl S217 S232 2646490 10.1097/00005650-198903001-00018

[B10] Cella D Yount S Rothrock N Gershon R Cook K Reeve B Ader D Fries JF Bruce B Rose M The Patient-Reported Outcomes Measurement Information System (PROMIS): progress of an NIH Roadmap cooperative group during its first two years Med Care 2007 45 5 Suppl 1 S3 S11 17443116 10.1097/01.mlr.0000258615.42478.55 PMC2829758

[B11] Herdman M Gudex C Lloyd A Janssen M Kind P Parkin D Bonsel G Badia X Development and preliminary testing of the new five-level version of EQ-5D (EQ-5D-5L) Qual Life Res 2011 20 10 1727 1736 21479777 10.1007/s11136-011-9903-x PMC3220807

[B12] Buist AS McBurnie MA Vollmer WM Gillespie S Burney P Mannino DM Menezes AM Sullivan SD Lee TA Weiss KB Jensen RL Marks GB Gulsvik A Nizankowska-Mogilnicka E BOLD Collaborative Research Group International variation in the prevalence of COPD (the BOLD Study): a population-based prevalence study Lancet 2007 370 9589 741 750 17765523 10.1016/S0140-6736(07)61377-4

[B13] ATS Committee on Proficiency Standards for Clinical Pulmonary Function Laboratories ATS statement: guidelines for the six-minute walk test Am J Respir Crit Care Med 2002 166 1 111 117 12091180 10.1164/ajrccm.166.1.at1102

[B14] Brooks SM Surveillance for respiratory hazards ATS News 1982 8 12 16

[B15] Borg GAV Psychophysical bases of perceived exertion Med Sci Sports Exerc 1982 14 5 377 381 7154893

[B16] Yount SE Choi SW Victorson D Ruo B Cella D Anton S Hamilton A Brief, valid measures of dyspnea and related functional limitations in chronic obstructive pulmonary disease (COPD) Value Health 2011 14 2 307 315 21402298 10.1016/j.jval.2010.11.009

[B17] Choi SW Victorson DE Yount S Anton S Cella D Development of a conceptual framework and calibrated item banks to measure patient-reported dyspnea severity and related functional limitations Value Health 2011 14 2 291 306 21402297 10.1016/j.jval.2010.06.001

[B18] Hinchcliff M Beaumont JL Thavarajah K Varga J Chung A Podlusky S Carns M Chang RW Cella D Validity of two new patient-reported outcome measures in systemic sclerosis: Patient-Reported Outcomes Measurement Information System 29-item Health Profile and Functional Assessment of Chronic Illness Therapy-Dyspnea short form Arthritis Care Res (Hoboken) 2011 63 11 1620 1628 22034123 10.1002/acr.20591 PMC3205420

[B19] Rabin R de Charro F EQ-5D: a measure of health status from the EuroQol Group Ann Med 2001 33 5 337 343 11491192 10.3109/07853890109002087

[B20] van Hout B Janssen MF Feng YS Kohlmann T Busschbach J Golicki D Lloyd A Scalone L Kind P Pickard AS Interim scoring for the EQ-5D-5L: mapping the EQ-5D-5L to EQ-5D-3L value sets Value Health 2012 15 5 708 715 22867780 10.1016/j.jval.2012.02.008

[B21] EuroQoL Group Crosswalk value sets for the EQ-5D-5Lhttp://www.euroqol.org/about-eq-5d/valuation-of-eq-5d/eq-5d-5l-value-sets.html

[B22] Cella D Riley W Stone A Rothrock N Reeve B Yount S Amtmann D Bode R Buysse D Choi S Cook K Devellis R DeWalt D Fries JF Gershon R Hahn EA Lai JS Pilkonis P Revicki D Rose M Weinfurt K Hays R PROMIS Cooperative Group The Patient-Reported Outcomes Measurement Information System (PROMIS) developed and tested its first wave of adult self-reported health outcome item banks: 2005-2008 J Clin Epidemiol 2010 63 11 1179 1194 20685078 10.1016/j.jclinepi.2010.04.011 PMC2965562

[B23] Liu H Cella D Gershon R Shen J Morales LS Riley W Hays RD Representativeness of the Patient-Reported Outcomes Measurement Information System Internet panel J Clin Epidemiol 2010 63 11 1169 1178 20688473 10.1016/j.jclinepi.2009.11.021 PMC2943555

[B24] PROMIS Scoring Manuals [http://www.assessmentcenter.net/Manuals.aspx]

[B25] Cohen J Statistical Power Analysis for the Behavioral Sciences 1988 2 New Jersey: Lawrence Erlbaum Associates

[B26] Brown CA Cheng EM Hays RD Vassar SD Vickrey BG SF-36 includes less Parkinson Disease (PD)-targeted content but is more responsive to change than two PD-targeted health-related quality of life measures Qual Life Res 2009 18 9 1219 1237 19714487 10.1007/s11136-009-9530-y PMC2759458

[B27] Hays RD Anderson R Revicki D Psychometric considerations in evaluating health-related quality of life measures Qual Life Res 1993 2 6 441 449 8161978 10.1007/BF00422218

[B28] Liang MH Larson MG Cullen KE Schwartz JA Comparative measurement efficiency and sensitivity of five health status instruments for arthritis research Arthritis Rheum 1985 28 5 542 547 4004963 10.1002/art.1780280513

[B29] Lix LM Keselman JC Keselman HJ Consequences of assumption violations revisited: A quantitative review of alternatives to the one-way analysis of variance F test Rev Educ Res 1996 66 4 579 619

[B30] Nunnally JC Bernstein IH Psychometric Theory 1994 3 New York: McGraw-Hill

[B31] Stavem K Reliability, validity and responsiveness of two multiattribute utility measures in patients with chronic obstructive pulmonary disease Qual Life Res 1999 8 1–2 45 54 10457737 10.1023/a:1026475531996

[B32] Wijnhoven HA Kriegsman DM Hesselink AE Penninx BW de Haan M Determinants of different dimensions of disease severity in asthma and COPD: pulmonary function and health-related quality of life Chest 2001 119 4 1034 1042 11296166 10.1378/chest.119.4.1034

[B33] Hesselink AE van der Windt DA Penninx BW Wijnhoven HA Twisk JW Bouter LM van Eijk JT What predicts change in pulmonary function and quality of life in asthma or COPD? J Asthma 2006 43 7 513 519 16939991 10.1080/02770900600856954

[B34] Hajiro T Nishimura K Tsukino M Ikeda A Oga T Izumi T A comparison of the level of dyspnea vs disease severity in indicating the health-related quality of life of patients with COPD Chest 1999 116 6 1632 1637 10593787 10.1378/chest.116.6.1632

[B35] Pickard AS Yang Y Lee TA Comparison of health-related quality of life measures in chronic obstructive pulmonary disease Health Qual Life Outcomes 2011 9 26 21501522 10.1186/1477-7525-9-26 PMC3096892

[B36] Rodriguez Gonzalez-Moro JM de Lucas RP Izquierdo Alonso JL Lopez-Muniz Ballesteros B Anton Diaz E Ribera X Martin A Impact of COPD severity on physical disability and daily living activities: EDIP-EPOC I and EDIP-EPOC II studies Int J Clin Pract 2009 63 5 742 750 19392924 10.1111/j.1742-1241.2009.02040.x

[B37] Rothrock NE Hays RD Spritzer K Yount SE Riley W Cella D Relative to the general US population, chronic diseases are associated with poorer health-related quality of life as measured by the Patient-Reported Outcomes Measurement Information System (PROMIS) J Clin Epidemiol 2010 63 11 1195 1204 20688471 10.1016/j.jclinepi.2010.04.012 PMC2943571

[B38] Janssen MF Pickard AS Golicki D Gudex C Niewada M Scalone L Swinburn P Busschbach J Measurement properties of the EQ-5D-5L compared to the EQ-5D-3L across eight patient groups: a multi-country study Qual Life Res 2013 22 7 1717 1727 23184421 10.1007/s11136-012-0322-4 PMC3764313

[B39] Pickard AS Wilke C Jung E Patel S Stavem K Lee TA Use of a preference-based measure of health (EQ-5D) in COPD and asthma Respir Med 2008 102 4 519 536 18180151 10.1016/j.rmed.2007.11.016

[B40] Punekar YS Rodriguez-Roisin R Sculpher M Jones P Spencer M Implications of chronic obstructive pulmonary disease (COPD) on patients’ health status: a western view Respir Med 2007 101 3 661 669 17197164 10.1016/j.rmed.2006.06.001

[B41] Antonelli-Incalzi R Imperiale C Bellia V Catalano F Scichilone N Pistelli R Rengo F Do GOLD stages of COPD severity really correspond to differences in health status? Eur Respir J 2003 22 3 444 449 14516133 10.1183/09031936.03.00101203

[B42] Norman GR Sloan JA Wyrwich KW Interpretation of changes in health-related quality of life: the remarkable universality of half a standard deviation Med Care 2003 41 5 582 592 12719681 10.1097/01.MLR.0000062554.74615.4C

[B43] Blinderman CD Homel P Billings JA Tennstedt S Portenoy RK Symptom distress and quality of life in patients with advanced chronic obstructive pulmonary disease J Pain Symptom Manage 2009 38 1 115 123 19232893 10.1016/j.jpainsymman.2008.07.006

[B44] Livneh H Antonak RF Psychosocial adaptation to chronic illness and disability: a primer for counselors J Couns Dev 2005 83 1 12 20

[B45] Revicki DA Kawata AK Harnam N Chen WH Hays RD Cella D Predicting EuroQol (EQ-5D) scores from the patient-reported outcomes measurement information system (PROMIS) global items and domain item banks in a United States sample Qual Life Res 2009 18 6 783 791 19472072 10.1007/s11136-009-9489-8 PMC2704290

