# Supplementary material for: Measuring health-related quality of life in chronic obstructive pulmonary disease: properties of the EQ-5D-5L and PROMIS-43 short form
Source: BMC Med Res Methodol. 2014 Jun 16;14:78. doi: 10.1186/1471-2288-14-78 (PMC4072614; doi:10.1186/1471-2288-14-78)
Supplement: Additional file 1 — Figure A1. Flow diagram of CONCERT patient recruitment and follow-up. Table A-1. Pathways for inclusion in CONCERT-CER Data Warehouse. Table A-2. Definition of tiers used to define sampling strata. [file 1471-2288-14-78-S1.pdf]

Additional File 1

Article title: Measuring health-related quality of life in chronic obstructive pulmonary disease: properties of the EQ-5D-5L and PROMIS-43 Short Form

Journal: ***BMC Medical Research Methodology***

Authors: Fang-Ju Lin, PhD, Todd A. Lee, PharmD, PhD, Jerry A. Krishnan, MD, PhD, Min J. Joo, MD, MPH, David H. Au, MD, MS, Shannon S. Carson, MD, MS, Suzanne Gillespie, MS, Ashley G. Henderson, MD, Peter K. Lindenauer, MD, MS, FACP, Mary Ann McBurnie, PhD, Richard A. Mularski, MD, MSHS, MCR, Edward T. Naureckas, MD, William M. Vollmer, PhD, A. Simon Pickard, PhD [on behalf of the CONCERT Consortium]

Seven U.S. clinical centers were involved in the CONCERT patient recruitment: Kaiser Permanente Northwest Region, VA Puget Sound Health Care System, University of Chicago, University of Illinois Hospital and Health Sciences System, University of Washington, Baystate Medical Center, and University of North Carolina at Chapel Hill. The CONCERT investigators developed a COPD Data Warehouse (CDW), containing comprehensive information from seven health care organizations on more than 220,000 patients with some indication of a chronic respiratory condition between 2006 and 2010. Since the focus of CONCERT work is on ***effectiveness*** research, it was critical from a scientific standpoint that the CDW include all or nearly all patients with COPD, including those with relatively mild disease. This comprehensive population will provide the backdrop against which results of CDW-based studies can be interpreted (i.e., this population serves as the denominator) and also provides the ability to select and analyze sub-populations of interest. It was also desirable to cover a substantial period of time for data collection to enable linking across patient healthcare encounters. Basic inclusion criteria for the CDW required that patients were aged 40 or greater as of 12/31/2006 and had at least one clinical visit between 2006 and 2010. In addition, patients had to meet at least one of six “COPD pathway” criteria (Table A-1). Patients who met these criteria had all available healthcare encounter data (COPD-related or not) included in the CDW.

In addition to the full CDW, CONCERT investigators recruited a sample of 1,206 patients from the CDW for in-person evaluations that included post-bronchodilator spirometry, six-minute walk test, and extensive patient-reported outcomes (dyspnea scores, quality of life, etc) (Figure A-1). This sample provided the ability to validate the CDW data and link it to disease severity, functional status, and other patient-centered outcomes and to accelerate patient enrollment into subsequent comparative effectiveness research studies. Patients were identified for recruitment based on sampling strata designed to compare how well each strata predict the identification of patients with COPD. Patients were assigned to the

highest level (i.e., higher likelihood of having COPD) strata for which they met criteria (see Table A-2). Patients were randomly selected from these strata for recruitment. In-person training on protocol implementation and data management was conducted at all sites before patient recruitment. Patients were excluded if they could not perform a spirometry test or could not participate due to cognitive impairment, frailty, acute illness, receiving hospice care or staying in long-term care facility, and issues related to geography, administration, procedure or communication. Overall, the study had a 36% response rate, calculating as the proportion of responders among participants who were actively contacted. All study participants were consented before participation.

Figure A-1. Flow diagram of CONCERT patient recruitment and follow-up

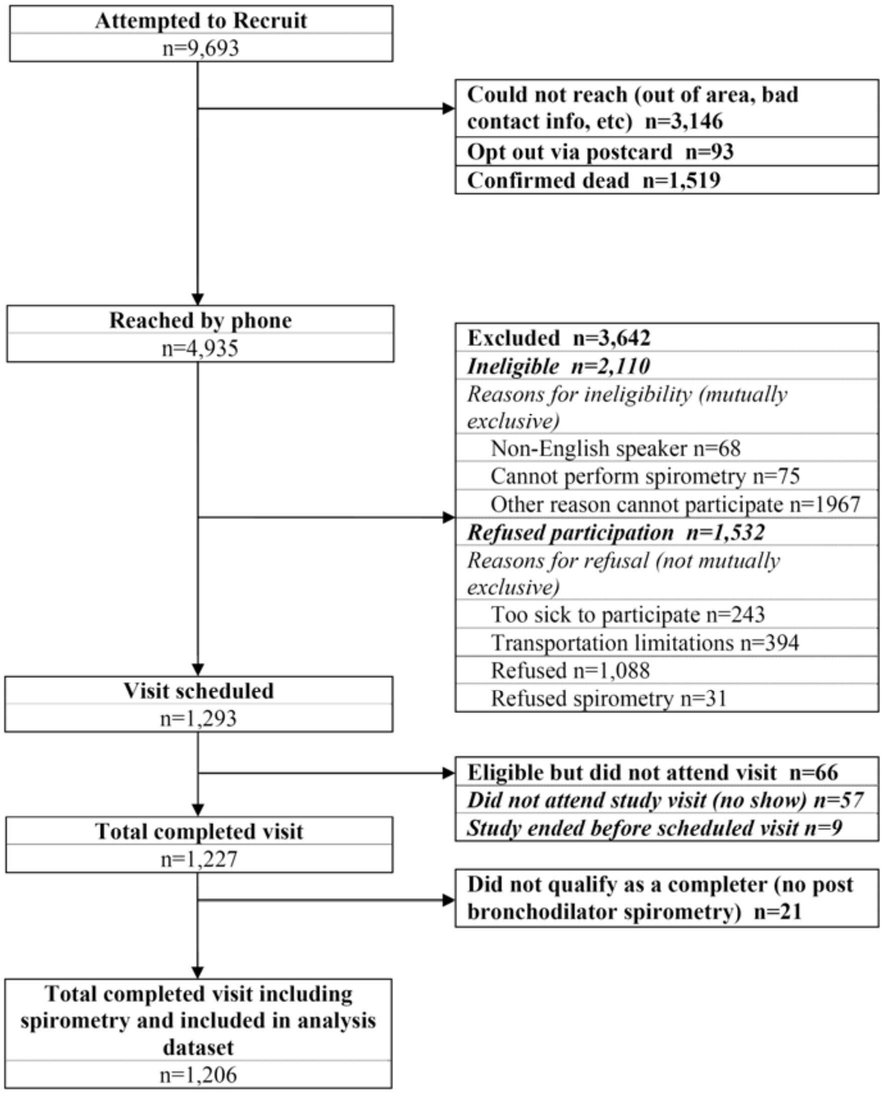

Table A-1. Pathways for inclusion in CONCERT-CER Data Warehouse

Pathway	Criteria
1. Outpatient COPD encounter	ICD-9 (or corresponding SNO-MED) codings:<br>491.xx – chronic bronchitis<br>492.xx – emphysema<br>493.2x – chronic obstructive asthma<br>496.xx – chronic airway obstruction
2. Inpatient COPD encounter	ICD-9 (or corresponding SNO-MED) codings:<br>490.xx – bronchitis, not specified as acute or chronic<br>491.xx – chronic bronchitis<br>492.xx – emphysema<br>493.2x – chronic obstructive asthma<br>494.xx – bronchiectasis<br>495.xx – extrinsic allergic alveolitis<br>496.xx – chronic airway obstruction
3. Hospitalized respiratory failure (primary) with secondary COPD coding	ICD-9 (or corresponding SNO-MED) codings:<br>518.81 – acute respiratory failure<br>518.82 – other pulmonary insufficiency not elsewhere classified<br>518.84 – acute and chronic respiratory failure
4. Problem List	Coded fields ICD-9 / variant "V" coding or SNO-MED) – COPD, emphysema, chronic bronchitis, chronic airway obstruction
5. PFT demonstrating likely obstructive lung disease	Evidence of pre or post-bronchodilator obstruction defined by an FEV1 / FVC ratio < 0.7
6. Documented COPD-related drug dispensing, order or medication listing	Beta-agonists by nebulized delivery<br>Beta-agonists, inhalers – long-acting<br>Ipatropium & tiotropium<br>Inhaled corticosteroid (ICS)<br>Combivent (ipratropium + albuterol)<br>Combination inhaled beta-agonists + ICS (e.g., Advair)

CER = comparative effectiveness research; FEV_1 = forced expiratory volume in 1 second; FVC = forced vital capacity; ICD-9 = International Classification of Diseases, ninth revision; ICS = inhaled corticosteroid; PFT = pulmonary function test; SNO-MED = Systematized Nomenclature of Medicine.

Table A-2. Definition of tiers used to define sampling strata

Tier	Pathway Criteria	Details
1	*At least one COPD path = 1*	Catch all for all the patients who didn't make it via other layers. We start from layer 6.
2	*Pathway 1 (Outpatient diagnosis code for COPD, including ED and urgent care visits)*	491.xx – chronic bronchitis; 492.xx – emphysema; 493.2x – chronic obstructive asthma; 496.xx – chronic airway obstruction, not elsewhere classified
	Pathway 4	Problem List (inclusive coded ICD-9 variant "V" coding or SNO-MED) – COPD, emphysema, chronic bronchitis, chronic airway obstruction
	Pathway 6 (Anticholinergics)	Medication dispensing, order or medication listing for ipratropium or tiotropium
3	*Pathway 2 (Inpatient visit with select COPD codes in any position)*	491.xx – chronic bronchitis; 492.xx – emphysema; 496.xx – chronic airway obstruction, not elsewhere classified
4	*Pathway 1(Outpatient diagnosis code for COPD in any position)*	491.xx – chronic bronchitis; 492.xx – emphysema; 493.2x – chronic obstructive asthma; 496.xx – chronic airway obstruction, not elsewhere classified
	PLUS	***PLUS***
	Pathway 2 (Inpatient visit with select COPD codes in any position)	491.xx – chronic bronchitis; 492.xx – emphysema; 496.xx – chronic airway obstruction, not elsewhere classified
5	*Pathway 4 (Problem List consistent with diagnosis of COPD)*	Problem List (inclusive coded ICD-9 variant "V" coding or SNO-MED) – COPD, emphysema, chronic bronchitis, chronic airway obstruction
	PLUS	***PLUS***
	Pathway 2 (Inpatient visit with select COPD codes in any position)	491.xx – chronic bronchitis; 492.xx – emphysema; 496.xx – chronic airway obstruction, not elsewhere classified. This includes Pathway 3 (Hospitalized respiratory failure [primary diagnosis] with secondary inpatient COPD coding).
	**OR**	
	Pathway 6 (Medications for COPD)	Beta-agonists by nebulized delivery, inhaled corticosteroid (ICS), Combivent (ipratropium + albuterol), combination inhaled beta-agonists + ICS (e.g., Advair), ipratropium and tioptropium, long-acting beta-agonist inhalers
	PLUS	***PLUS***
	Pathway 1 (Outpatient diagnosis code for COPD in any position)	491.xx – chronic bronchitis; 492.xx – emphysema; 493.2x – chronic obstructive asthma; 496.xx – chronic airway obstruction, not elsewhere classified
	PLUS	***PLUS***
	Pathway 2 (Inpatient visit with select COPD codes in any position	491.xx – chronic bronchitis; 492.xx – emphysema; 496.xx – chronic airway obstruction, not elsewhere classified
	**OR**	
	Pathway 6 (Medications for COPD)	Beta-agonists by nebulized delivery, inhaled corticosteroid (ICS), Combivent (ipratropium + albuterol), combination inhaled beta-agonists + ICS (e.g., Advair), ipratropium and tioptropium, long-acting beta-agonist inhalers
	PLUS	***PLUS***
	Pathway 4 (Problem List consistent with diagnosis of COPD)	Problem List (inclusive coded ICD-9 variant "V" coding or SNO-MED) – COPD, emphysema, chronic bronchitis, chronic airway obstruction
	PLUS	***PLUS***
	Pathway 2 (Inpatient visit with select COPD codes in any position	491.xx – chronic bronchitis; 492.xx – emphysema; 496.xx – chronic airway obstruction, not elsewhere classified
6	*Pathway5 (Spirometry consistent with COPD)*	PFTs consistent with COPD defined as ratio of FEV1 to FVC <0.7 (either pre or post bronchodilator spirometry can be used)
	PLUS	***PLUS***
	At least one or more encounters from Pathway 1, Pathway 2 and Pathway 3 (subset of Pathway 2)	491.xx – chronic bronchitis; 492.xx – emphysema; 493.2x – chronic obstructive asthma; 496.xx – chronic airway obstruction, not elsewhere classified; 490.xx – bronchitis, not specified as acute or chronic; 494.xx – bronchiectasis; 495.xx – extrinsic allergic alveolitis
	**OR**	
	Pathway 5 (Spirometry consistent with COPD)	PFTs consistent with COPD defined as ratio of FEV1 to FVC <0.7
	PLUS	***PLUS***

	Pathway 4 (Problem List consistent with diagnosis of COPD)	Problem List (inclusive coded ICD-9 variant "V" coding or SNO-MED) – COPD, emphysema, chronic bronchitis, chronic airway obstruction

ED = emergency department; FEV_1 = forced expiratory volume in 1 second; FVC = forced vital capacity; ICD-9 = International Classification of Diseases, ninth revision; ICS = inhaled corticosteroid; PFT = pulmonary function test; SNO-MED = Systematized Nomenclature of Medicine.
